# Episiotomy and obstetric outcomes among women living with type 3 female genital mutilation: a secondary analysis

**DOI:** 10.1186/s12978-016-0242-9

**Published:** 2016-10-10

**Authors:** Maria I. Rodriguez, Armando Seuc, Lale Say, Michelle J. Hindin

**Affiliations:** 1 Department of Reproductive Health and Research, World Health Organization, Avenue Appia 20, 1211 Geneva, Switzerland; 2 Department of Obstetrics and Gynecology, Oregon Health & Science University, Portland, OR USA

**Keywords:** Female genital mutilation, Episiotomy, FGM, Circumcision, Obstetrics

## Abstract

**Background:**

To investigate the association between type of episiotomy and obstetric outcomes among 6,187 women with type 3 Female Genital Mutilation (FGM).

**Methods:**

We conducted a secondary analysis of women presenting in labor to 28 obstetric centres in Burkina Faso, Ghana, Kenya, Nigeria, Senegal and Sudan between November 2001 and March 2003. Data were analysed using cross tabulations and multivariable logistic regression to determine if type of episiotomy by FGM classification had a significant impact on key maternal outcomes. Our main outcome measures were anal sphincter tears, intrapartum blood loss requiring an intervention, and postpartum haemorrhage.

**Results:**

Type of episiotomy performed varied significantly by FGM status. Among women without FGM, the most common type of episiotomy performed was posterior lateral (25.4 %). The prevalence of the most extensive type of episiotomy, anterior and posterior lateral episiotomy increased with type of FGM. Among women without FGM, 0.4 % had this type of episiotomy. This increased to 0.6 % for women with FGM Types 1, 2 or 4 and to 54.6 % of all women delivering vaginally with FGM Type 3. After adjustment, women with an anterior episiotomy, (AOR = 0.15 95 %; CI 0.06–0.40); posterior lateral episiotomy (AOR = 0.68 95 %; CI 0.50–0.94) or both anterior and posterior lateral episiotomies performed concurrently (AOR = 0.21 95 % CI 0.12–0.36) were all significantly less likely to have anal sphincter tears compared to women without episiotomies. Women with anterior episiotomy (AOR = 0.08; 95%CI 0.02–0.24), posterior lateral episiotomy (AOR = 0.17 95 %; CI 0.05–0.52) and the combination of the two (AOR = 0.04 95 % CI 0.01–0.11) were significantly less likely to have postpartum haemorrhage compared with women who had no episiotomy.

**Conclusions:**

Among women living with FGM Type 3, episiotomies were protective against anal sphincter tears and postpartum haemorrhage. Further clinical and research is needed to guide clinical practice of when episiotomies should be performed.

## Plain English summary

Female genital mutilation (FGM) encompasses a range of procedures that damage and change women’s external genitalia. More than 200 million girls and women have been subjected to FGM, and an estimated three million girls are at risk every year. FGM has significant effects on women’s health, especially during pregnancy and delivery. There is very little information available for health care providers to help provide evidence based care for women living with FGM, and minimize obstetric risks. We looked at how episiotomy, an incision to extend the vaginal opening during birth, varied by FGM status. We also looked at whether type of episiotomy improved maternal health outcomes. We found that women living with FGM were more likely to have the most extensive types of episiotomies performed. Our findings suggest that anterior episiotomy, to release scar tissue, may reduce some obstetrical risk among women with the most extensive type of FGM. We need more information to help women and providers decide when is the best time to provide defibulation during pregnancy.

## Background

Female Genital Mutilation (FGM) includes a range of procedures involving partial or total removal of the external female genitalia for non-therapeutic reason [1]. The World Health Organization (WHO) has defined four types of FGM (Table 1). The procedures performed vary by country, and range from partial or total removal of the clitoris (Type 1) to narrowing of the vaginal opening by the removal and suturing of the labia (Type 3). Type 4 consists of all other harmful procedures to the female genitalia for non-medical purposes, for example, pricking, piercing, incising, scraping and cauterisation.

**Table 1:** WHO classification of Female Genital Mutilation

Type I : Partial or total removal of the clitoris^a^ and/or the prepuce (clitoridectomy)
Type Ia: Removal of the clitoral hood or prepuce only
Type Ib: Removal of the clitoris^a^ with the prepuce
Type II: Partial or total removal of the clitoris^a^ and the labia minora, with or without excision of the labia majora (excision)
Type IIa: Removal of the labia minora only
Type IIb: Partial or total removal of the clitoris^a^ and the labia minora
Type IIc: Partial or total removal of the clitoris^a^, the labia minora and the labia majora
Type III: Narrowing of the vaginal orifice with creation of a covering seal by cutting and apposition the labia minora and/or the labia majora, with or without excision of the clitoris (infibulation)
Type IIIa: Removal and apposition of the labia minora
Type IIIb: Removal and apposition of the labia majora
Type IV: Unclassified
All other harmful procedures to the female genitalia for non-medical purposes, for example, pricking, piercing, incising, scraping and cauterisation

^a^When total removal of the clitoris is reported, it refers to the total removal of the glans of the clitoris

The impact of FGM on obstetric outcomes has been investigated in several studies [2–4]. Compared to women without FGM, women with FGM have an increased risk of episiotomy, caesarean delivery, haemorrhage, extended maternal hospital stay, infant resuscitation, and inpatient perinatal death [3]. The risk of adverse obstetric outcomes varies by FGM type, with the most extensive forms of FGM being associated with the greatest risk [3, 5]. Women with Type 3 FGM have been shown to have increased risk of episiotomy, caesarean delivery, postpartum haemorrhage and stillbirth [3]. There is an urgent need for evidence on how to minimize the negative perinatal consequences for women living with FGM [6, 7]. The majority of existing recommendations for obstetric practice in this population are based on expert opinion [6]. New guidelines from the WHO examine the evidence for optimizing the health care management of women living with FGM [8]. Topics included reflect a broad range of health care needs including: female sexual health, mental health, information & education needs for women and providers, as well as defibulation. Improved data to guide defibulation practices was identified as a research priority by the WHO.

The scar tissue from FGM, in particular with Type 3, narrows the vaginal introitus, and is thought to increase the risk for obstructed labour and extensive perineal lacerations [9, 10]. Prolonged labour is a risk factor for postpartum haemorrhage [11]. Anterior episiotomy (or defibulation) to release the scar tissue is commonly performed, but when a circumcised woman presents in labour, the optimal type of episiotomy and time to perform it is not known. Performing the procedure early in labour requires anesthesia, and may increase risk of intrapartum bleeding, as the incision would be irritated by subsequent cervical exams. [9] Delaying the procedure until immediately prior to delivery may increase the risk of postpartum haemorrhage due to obstructed labour.

Episiotomy is the surgical enlargement of the vaginal opening due to a perineal incision [5, 12]. Seven different types of episiotomies are reported in the literature, although only anterior, mediolateral and midline posterior are commonly used [13]. Among women without FGM, anterior, mediolateral and midline posterior episiotomies are typically performed. A posterior lateral episiotomy may also be referred to as a “J-shaped” episiotomy [13]. Anterior episiotomy, or defibulation, is the opening of the scar associated with FGM, most commonly used with women living with FGM Type 3 [13]. It is frequently performed during labour, to allow for cervical exams and to prevent obstructed labour [14, 15]. Anterior episiotomy may be performed alone, or in combination with midline posterior or posterior lateral episiotomies. A provider may choose to only perform a midline posterior or posterior lateral episiotomy as well, to avoid incising the scar tissue anteriorly. The decision of what type of episiotomy to perform is typically based on provider training and preference. Episiotomy is not without risks: it is associated with increased risk of pain, perineal trauma (extensive lacerations), need for suturing, and healing complications [12]. It is likely that the more extensive the episiotomy performed, the greater the risk of maternal harm.

There is scant evidence to guide episiotomy practice among women living with FGM [6, 16]. All existing guidelines are based on expert opinion with respect to episiotomy practice and FGM. The Royal College of Obstetricians and Gynaecologists recommends that intrapartum episiotomy in women with FGM be performed if inelastic scar tissue prevents progress. In general, existing guidelines advise a low threshold for performing episiotomy, despite the absence of studies on the real benefits of episiotomy with each type of FGM [6, 17]. No evidence exists to guide the type or timing of episiotomy to perform.

The objective of this study is to investigate the association between type of episiotomy and obstetric outcomes among women with living with FGM Type 3. We examine whether episiotomy improves maternal outcomes including anal sphincter tears, intrapartum blood loss requiring intervention, and postpartum haemorrhage.

## Methods

The WHO previously conducted an international, multicentre study examining obstetric outcomes in women by FGM status. The cohort contained women without FGM, as well as women with FGM, categorized by the WHO classification system. Previous papers have reported on the risks of different obstetric outcomes for both the woman and the neonate, as well as estimated costs to the health system [3, 18]. In this sub analysis, we focus on the association between type of episiotomy and maternal outcomes in women with FGM Type 3.

Women who presented for singleton delivery at 28 obstetric centres in Burkina Faso, Ghana, Kenya, Nigeria, Senegal and Sudan between November 2001 and March 2003 were screened for study eligibility. Women with multiple gestations, or presenting for elective caesarean delivery or in advanced labour (unable to complete a pelvic exam prior to delivery) were excluded from the study, along with women who were unwilling or unable to give informed consent. Women and their infants were then followed until time of maternal discharge from the hospital. All participants provided informed consent prior to enrolment. Institutional review boards at all participating hospitals and the World Health Organization (WHO) Secretariat Committee on Research Involving Human Subjects gave ethics approval.

We used descriptive statistics and bivariate measures of association to describe the study population and the population of women by type of FGM. Bivariate and multivariable logistic regression models were used to examine the association of type of episiotomy and maternal outcomes (anal sphincter tears, intrapartum blood loss requiring intervention, and postpartum haemorrhage) among women with type 3 FGM.

### Study population

We included only women having a vaginal delivery; this included normal vaginal delivery, assisted operative delivery (forceps or vacuum) and assisted breech delivery. Women giving birth by caesarean were excluded. Participants had an antepartum examination of the external genitalia, by a trained study midwife, to determine whether or not they had undergone FGM. If they had FGM, the type was categorized according to the WHO classification system (Table 1). The pelvic exam also included an assessment of outlet obstruction: the dimension of the introitus was evaluated by fingerbreadths. For the analysis of the association between episiotomy and maternal health outcomes, we limited our sample to women who were living with FGM Type 3 with data on episiotomy status.

### Study variables

Our key independent variable for analysis was episiotomy type. If an episiotomy was performed, the study investigator recorded the type. Episiotomy was classified as follows: no episiotomy, anterior (deinfibulation), posterior lateral, and anterior with simultaneous posterior lateral episiotomy. The dimension of the introitus was assessed by finger breadths and coded as one, two, three, or more than three fingerbreadths. For the multivariable models, we included the following demographic characteristics of the woman: her age, place of residence (urban/rural), socioeconomic status (low, medium, high) and level of education.

Three maternal health outcomes served as our dependent variables—anal sphincter tears, intrapartum blood loss requiring an intervention, and post partum haemorrhage. Degree of tear was included as a dichotomous variable, with comparing more extensive lacerations (anal sphincter tears, also called 3^rd^ and 4^th^ degree obstetric tears) to no tear or 1st or 2^nd^ degree tears. Intrapartum blood loss was dichotomized comparing women who required an intervention (e.g uterotonics, dilation and curettage, transfusion) to those who did not. Postpartum haemorrhage, blood loss occurring within 24 h of delivery, was coded as a binary variable using the standard threshold of exceeding 500 ml [11].

### Models

We examined the association between episiotomy type among women living with FGM Type 3 and each of the following outcomes—anal sphincter tears, intrapartum bleeding requiring intervention, and postpartum haemorrhage. Each type of episiotomy was compared with no episiotomy. Theoretically relevant model covariates included parity, pelvic introitus width, age, socioeconomic status, and education level. Initially we planned to enter the covariates in blocks—obstetric factors, sociodemographic factors and then the combination for fully adjusted models. However, the adjustment variables had minimal impact so we present only the unadjusted and then fully adjusted models. Odds ratios (OR) with 95 % confidence intervals were assessed for each of the three maternal outcomes. As the data were clustered in the 28 centres, robust standard errors were used to adjust for this clustering [19].

## Results

Table 2 shows the characteristics of the sample population overall, and by type of FGM. 26,640 women were included—6,744 who had no FGM, 6,211 with Type 3 FGM, and 13,685 with any other type of FGM (Types 1, 2 and 4; Table 2). The majority had undergone FGM (74.7 %) and were multiparous (95.8 %). The mean age was 26, and the majority lived in an urban setting (Table 2). The majority of births were spontaneous vaginal deliveries (90.0 %) with assisted vaginal delivery (vacuum or forceps) accounting for 2.7 % of births, and assisted breech deliveries 1.1 %. Compared to women who had either no FGM or FGM Types 1, 2 and 4, women with FGM Type 3 were significantly older, more likely to live in urban areas, have more education, medium SES and to be living in Sudan. These women were also significantly more likely to have an anterior/posterior episiotomy, and significantly less likely have anal sphincter, intrapartum and postpartum haemorrhage. We then analysed the characteristics of our population by type of episiotomy performed (Table 3). Compared to women with no episiotomies, women with anterior episiotomies were significantly older (27.4 vs 30.1) and of urban residence (76.1 % vs 67.6 %). Women with posterior lateral episiotomies were significantly more likely to have no education (22.5 % vs 39 %) than women without episiotomies. And lastly, women with the most extensive episiotomy type (anterior and midline posterior) were found to be significantly more likely to be of urban residence (72.4 % vs 67.6 %) and significantly less likely to be of low socioeconomic status (9 % vs 37.9 %).

**Table 2:** Sociodemographic and delivery characteristics by FGM type

	Full sample (*n* = 26,640)	No FGM (*n* = 6,696)	Type 3 FGM (*n* = 6,187)	Other FGM (*n* = 13,591)
FGM status (%)	24.0	25.2	23.4	51.3
Episiotomy (%)^a, b^				
None	57.3	71.1	13.8	70.4
Anterior	6.9	2.5	18.0	4.0
Posterior Lateral	21.5	25.4	12.3	23.7
Anterior and Posterior	13.6	0.6	54.5	1.3
Other	0.7	0.4	1.2	0.6
Age (mean) ^a, b^	26.3	25.1	27.1	26.5
Urban Residence ^a, b^	62.7	59.3	71.9	60.3
Education (%) ^a, b^				
No education	31.8	34.3	16.6	37.5
Non-formal	8.0	5.6	4.0	11.0
Primary	26.7	32.2	26.6	24.1
Secondary	25.0	23.1	38.4	19.9
Tertiary	8.4	4.8	14.4	7.5
Socioeconomic status (%)^a, b^				
Low	35.6	38.2	16.7	42.8
Medium	61.0	58.8	79.6	53.7
High	3.4	3.0	3.7	3.5
Country (%) ^a, b^				
Burkina Faso	17.0	13.2	9.3	22.5
Ghana	10.9	25.7	0.5	8.4
Kenya	18.9	8.9	0.6	31.9
Nigeria	14.6	23.3	6.4	14.1
Senegal	12.5	10.2	0.4	18.4
Sudan	26.5	18.7	82.7	4.8
Any Previous Births (%) ^a^	64.1	60.1	65.0	65.7
Pelvic introitus width ^a, b^ (mean fingerbreadths)	2.46	2.58	2.39	2.49
Anal sphincter tear (3^rd^ or 4^th^ Degree) (%)^a, b^	7.5	8.6	1.9	9.5
Intrapartum Blood Loss (requiring intervention) (%) ^a, b^	1.4	1.4	0.6	1.7
Postpartum haemorrhage (%)^a, b^	6.2	4.8	3.4	8.2

^a^ Type 3 statistically different from No FGM; ^b^ Type 3 statistically different from other FGM types

**Table 3:** Demographic characteristics of women with type 3 FGM by type of episiotomy

	No episiotomy	Anterior	Posterior lateral	Anterior & posterior	Other
	(*n* = 857)	(*n* = 1115)	(*n* = 763)	(*n* = 3377)	(*n* = 75)
Age (mean)	27.4	30.1^a^	24.9^a^	26.6^a^	21.8^a^
Urban Residence	67.6	76.1^a^	68.0	72.4^a^	76.0
Education (%)					
No education	39.0	18.0^a^	22.5^a^	9.0^a^	24.0
Non-formal	9.1	2.3	7.0	1.8	41.3
Primary	22.7	32.1	24.4	26.1	28.0
Secondary	21.7	41.1	35.0	43.3	4.0
Tertiary	7.5	6.5	11.1	19.8	2.7
Socioeconomic status (%)					
Low	37.9	14.1^a^	28.2^a^	9.0^a^	38.7
Medium	59.2	83.1	66.6	87.0	61.3
High	2.9	2.8	5.2	4.0	0.0
Country (%)					
Burkina Faso	31.5	5.9^a^	26.9^a^	0.8^a^	5.3^a^
Ghana	2.7	0.0	0.8	0.1	0.0
Kenya	2.7	0.4	0.9	0.2	1.3
Nigeria	22.5	0.8	11.3	1.3	90.1
Senegal	1.4	0.0	1.8	0.0	0.0
Sudan	39.2	92.9	58.3	97.7	2.7
Any Previous Births (%)	72.7	91.7^a^	53.0^a^	57.4^a^	42.7^a^

^a^Statistically different from “no episiotomy”

Women with FGM Type 3 had significantly narrower introituses when compared with women without FGM or with other types of FGM (mean of 2.37 fingers compared with 2.56 and 2.45, *p* < 0.001). Width of pelvic introitus was associated with episiotomy performed among women with FGM Type 3; women with more narrow introituses were significantly more likely to have an episiotomy. The analysis sample was limited to the 6,187 women who had FGM Type 3 with data on episiotomy status.

We first investigated whether type of episiotomy performed reduced risk of anal sphincter tear (3^rd^ or 4^th^ degree obstetric laceration) (Table 4). As there is minimal difference between the unadjusted and adjusted models, we present the adjusted results. Among women with FGM type 3, anterior, posterior lateral and anterior with posterior lateral episiotomy significantly decreased the odds of an anal sphincter tear. Compared with no episiotomy, anterior episiotomy had a stronger protective effect against anal sphincter tears (AOR = 0.15; 95 % CI 0.05–0.45) than posterior lateral (AOR = 0.66; 95 % CI 0.55–0.80) or both anterior and posterior lateral episiotomies performed concurrently (AOR = 0.21; 95 % CI 0.11–0.37).

**Table 4:** Unadjusted and adjusted odds ratios of anal sphincter tear among women with FGM Type 3 by episiotomy type

	Model 1: Unadjusted odds ratios (95 % CI)	Model 2: Adjusted odds ratio (95 % CI)
Episiotomy type		
None (comparison)	1.00	1.00
Anterior	0.11 (0.05–0.25)***	0.15 (0.06–0.40)***
Posterior lateral	0.72 (0.48–1.07)	0.68 (0.50–0.94)*
Anterior & Posterior lateral	0.18 (0.11–0.31)***	0.21 (0.12–0.36)***
Other	0.73 (0.09–5.87)	0.62 (0.04–6.4)
Obstetric characteristics		
Parity	--	0.64 (0.40–1.02)
Pelvic introitus	--	1.19 (0.89–1.60)
Demographic characteristics		
Age	--	0.98 (0.94–1.02)
Education	--	0.96 (0.73–1.26)
SES	--	0.71 (0.30–1.67)

***p ≤ 0.001, **p ≤ 0.01 ***p ≤ 0.05

Adjusted for clustering at the centre level (*n* = 28)

Note: Pelvic introitus assessed by fingerbreadths

With respect to postpartum haemorrhage (Table 5), among women with Type 3 FGM, all types of episiotomy were significantly associated with decreased odds of excessive bleeding postpartum (Table 5). Compared with no episiotomy, anterior episiotomy (AOR = 0.08; 95%CI 0.02–0.35), posterior lateral (AOR = 0.16; 95 % CI 0.04–0.67) and the combination of the two (AOR = 0.04; 95 % CI 0.02–0.09) had a protective effect against postpartum haemorrhage.

**Table 5:** Unadjusted and adjusted odds ratios of postpartum haemorrhage among women with FGM Type 3, by episiotomy type

	Model 1: Unadjusted odds ratio (95 % CI)	Model 2: Adjusted odds ratio (95 % CI)
Episiotomy type		
None (comparison)	1.00	1.00
Anterior	0.08 (0.02–0.26)***	0.08 (0.02–0.24)***
Posterior lateral	0.20 (0.07–0.60)***	0.17 (0.05–0.52)**
Anterior & Posterior lateral	0.05 (0.02–0.12)***	0.04 (0.01–0.11)***
Other	0.07 (0.02–0.23)***	0.06 (0.02–0.20)***
Obstetric characteristics		
Parity	--	0.56 (0.29–1.08)
Pelvic introitus	--	0.72 (042–1.24)
Demographic characteristics		
Age	--	1.02 (0.97–1.08)
Education	--	1.15 (0.77–1.71)
SES	--	0.82 (0.45–1.49)

***p ≤ 0.001, **p ≤ 0.01 ***p ≤ 0.05

Adjusted for clustering at the centre level (*n* = 28)

Note: Pelvic introitus assessed by fingerbreadths

We then examined the association between type of episiotomy and risk of intrapartum bleeding requiring intervention (Table 6). Among women with Type 3 FGM, no significant association was seen between anterior or posterior lateral episiotomy and odds of intrapartum bleeding. There was a statistically significant protective association between the combination of the two types of episiotomy, anterior and posterior lateral concurrently was observed (AOR = 0.03; 95 % CI 0.01–0.08).

**Table 6:** Unadjusted and adjusted odds ratio of intrapartum haemorrhage among women with FGM Type 3 by episiotomy type

	Model 1: Unadjusted odds ratio (95 % CI)	Model 2: Adjusted odds ratio (95 % CI)
Episiotomy type		
None (comparison)	1.00	1.00
Anterior	0.07 (0.004–1.2)	0.08 (0.005–1.31)
Posterior lateral	0.35 (0.07–1.68)	0.33 (0.05–2.14)
Anterior & Posterior lateral	0.03 (0.005–0.21)***	0.03 (0.004–0.27)***
Other	1.04 (0.25–4.34)	0.91 (0.12–7.04)
Obstetric characteristics		
Parity	--	0.57 (0.23–1.43)
Pelvic introitus	--	1.18 (0.53–2.34)
Demographic characteristics		
Age	--	1.00 (0.93–1.07)
Education	--	0.91 (0.60–1.39)
SES	--	1.08 (0.32–3.63)

***p ≤ 0.001, ***p ≤ 0.05

Adjusted for clustering at the centre level (n = 28)

Note: Pelvic introitus assessed by fingerbreadths

## Discussion

### Main findings

Our study suggests that among women with Type 3 FGM anterior episiotomy in labour is protective against anal sphincter tears and postpartum haemorrhage, and does not have a significant effect on intrapartum bleeding that required an intervention. A protective effect was seen with all types of episiotomy and anal sphincter tears and postpartum haemorrhage among women with FGM Type 3. Only concurrent anterior and posterior lateral episiotomies were associated with decreased odds of intrapartum blood loss requiring an intervention: this likely reflects the timing of when the different types of episiotomy occurred.

### Strengths and limitations

Our study should be interpreted with the following limitations in mind. A key limitation is that the indication for episiotomy was not recorded; episiotomy may have been performed for a specific medical indication such as obstructed labour or foetal distress, or done routinely based on provider preference. Timing of episiotomy is also not known, and this may have an impact on study outcomes. For example, the protective effect of anterior and posterior lateral episiotomy observed may be due to differences in timing of when providers performed episiotomies. If anterior episiotomies were differentially performed earlier in labour than other types, there would be greater length of time for bleeding to occur intrapartum.

Another limitation of our study is that it only includes facility based deliveries; women who delivered in the community are omitted. This biases our results towards the null, as this population may have worse outcomes. Additionally, women presenting for scheduled caesarean delivery were not eligible for study participation. Information regarding the indication for the caesarean would be of benefit in interpreting these findings.

While the full sample includes over 26,000 women across six African countries, it is important to note that the majority of women in our analytic sample (*n* = 6,211) with Type 3 FGM (82.7 %) came from Sudan. This affects the generalizability of our results. While we adjusted our models to account for data clustering by centre or facility, obstetric practices and medical training are thought to vary widely by country and facilities clustering does not fully account for this unobserved heterogeneity Currently FGM is not included in the curriculum of most medical and midwifery training, and recommendations regarding clinical management are not widely known [6]. Provider education regarding the appropriate management and clinical care of women living with FGM is essential to optimizing care. Strengths of our study include the relatively large analytic sample size of women living with Type 3 FGM. To our knowledge, no other study has provided evidence on the distribution of type of episiotomy by FGM classification or how this may impact maternal outcomes.

### Interpretation

Our study is consistent with previous evidence demonstrating that women with FGM have increased rates of episiotomy [3]. To date, episiotomy practice among women with FGM has been guided by expert opinion and provider preference. We provide new information on the association between type of episiotomy and key maternal outcomes (anal sphincter tear, intrapartum and postpartum bleeding) among women with Type 3 FGM. Our analysis demonstrates that episiotomy may reduce the odds of three poor obstetric outcomes; however, the risk of episiotomy needs to be also considered. Episiotomy is painful, and may result in infection, perineal trauma or healing complications [12]. Performing the smallest episiotomy necessary to achieve maternal or foetal gain is a reasonable clinical approach, however our data show that women living with FGM are more likely to have the most extensive type of episiotomy (anterior with concurrent posterior lateral episiotomy).

Working with providers to train them in the specific and evidence based care of women living with FGM is essential to mitigating the consequences of FGM [6, 8]. To achieve this, education on FGM needs to be incorporated into the curriculum of nursing, midwifery and medical programs. Additionally, clinical research is needed to investigate the impact of interventions in improving health outcomes for women, both during and outside of pregnancy [8].

## Conclusion

The objective of our study was to investigate the association between type of episiotomy and obstetric outcomes including anal sphincter tears, and intrapartum blood loss requiring an intervention, and postpartum hemorrhage among women with living with FGM Type 3. We found that all types of episiotomies are protective against these outcomes. Given the risks associated with episiotomy however, the smallest episiotomy needed should be utilized. Currently women living with FGM Type 3 are significantly more likely to have the most extensive type of episiotomy, with both an anterior and posterior incision. There is not strong data to support this clinical practice.

More data are needed to guide the medical care of women living with FGM. Evidence to inform when (antenatal or during labour) anterior episiotomy or deinfibulation is performed is urgently needed. Research to identify when episiotomy should be performed and for which women living with FGM is needed. Anterior episiotomy, or defibulation in pregnancy, at the first and at the second stage of labour, should be prospectively compared for blood loss, rate of episiotomy, perineal tear, demand for reinfibulation, and acceptance and satisfaction with deinfibulation for women. Provider training to improve the obstetrical care of women with FGM is also needed.

## Abbreviations

FGM: Female genital mutilation
WHO: World Health Organization

## References

[1] UNAIDS U, UNECA, UNESCO, UNFPA, UNHCHR, UNHCR, UNICEF, UNIFEM, WHO (2008). Eliminating female genital mutilation: an interagency statement.

[2] Berg RC, Odgaard-Jensen J, Fretheim A, Underland V, Vist G (2014). An updated systematic review and meta-analysis of the obstetric consequences of female genital mutilation/cutting. Obstet Gynecol Int.

[3] WHO study group on female genital mutilation and obstetric outcome: WHO collaborative prospective study in six African countries. Lancet. 2006;367:1835–41. 10.1016/S0140-6736(06)68805-3 16753486

[4] WHO (2000). A Systematic Review of the Health Complications of Female Genital Mutilation including Sequelae in Childbirth.

[5] WHO. Managing complications in pregnancy and childbirth: a guide for midwives and doctors. Geneva: World Health Organization; 2003.

[6] Abdulcadir J RM, Say L. Research gaps in the care of women with Female Genital Mutilation: an analysis. BJOG. 2014. in press 10.1111/1471-0528.13217 25514892

[7] Balogun OO, Hirayama F, Wariki WM, Koyanagi A, Mori R (2013). Interventions for improving outcomes for pregnant women who have experienced genital cutting. Cochrane Database Syst Rev.

[8] WHO (2016). WHO guidelines on the management of health complications from female genital mutilation.

[9] Rushwan H (2000). Female genital mutilation (FGM) management during pregnancy, childbirth and the postpartum period. Int J Gynaecol Obstet.

[10] De Silva S (1989). Obstetric sequelae of female circumcision. Eur J Obstet Gynecol Reprod Biol.

[11] Sheiner E, Sarid L, Levy A, Seidman DS, Hallak M (2005). Obstetric risk factors and outcome of pregnancies complicated with early postpartum hemorrhage: a population-based study. J Matern Fetal Neonatal Med.

[12] Carroli G, Mignini L. Episiotomy for vaginal birth. Cochrane Database Syst Rev. 2009:CD000081. 10.1002/14651858.CD000081.pub2 PMC4175536 19160176

[13] Kalis V, Laine K, de Leeuw JW, Ismail KM, Tincello DG (2012). Classification of episiotomy: towards a standardisation of terminology. BJOG.

[14] Shaw E. Female circumcision. What kind of maternity care do circumcised women need? Am J Nurs. 1985:684–7 3847258

[15] WHO. Management of pregnancy, childbirth and the postpartum period in the presence of female genital mutilation. Report of a WHO technical consultation. Geneva: World Health Organization; 1997.

[16] WHO (2001). Female Genital Mutilation. Integrating the prevention and the management of the health complications into the curricula of nursing and midwifery.

[17] Female Genital Mutilation and its Management: RCOG; 2015 July 2015.

[18] Bishai D, Bonnenfant YT, Darwish M, et al. Estimating the obstetric costs of female genital mutilation in six African countries. Bull World Health Organ. 2010;(4)88:281–8. 10.2471/BLT.09.064808 PMC2855596 20431792

[19] Diggle PJ HP, Liang K-Y, Zeger SL (2002). Analysis of longitudinal data.

